# Impella CP Implantation during Cardiopulmonary Resuscitation for Cardiac Arrest: A Multicenter Experience

**DOI:** 10.3390/jcm10020339

**Published:** 2021-01-18

**Authors:** Vassili Panagides, Henrik Vase, Sachin P. Shah, Mir B. Basir, Julien Mancini, Hayaan Kamran, Supria Batra, Marc Laine, Hans Eiskjær, Steffen Christensen, Mina Karami, Franck Paganelli, Jose P. S. Henriques, Laurent Bonello

**Affiliations:** 1 Intensive Care Unit, Department of Cardiology, Assistance Publique-Hôpitaux de Marseille, Hôpital Nord, Aix-Marseille University, 13015 Marseille, France; vassili.panagides@gmail.com (V.P.); Marc.LAINE@ap-hm.fr (M.L.); franck.paganelli@ap-hm.fr (F.P.); 2 Mediterranean Association for Research and Studies in Cardiology (MARS Cardio), 13015 Marseille, France; 3 Centre for CardioVascular and Nutrition Research (C2VN), INSERM 1263, INRA 1260, 13015 Marseille, France; 4 Department of Cardiology, Aarhus University Hospital, 8200 Aarhus, Denmark; henrikvase@dadlnet.dk (H.V.); heis@dadlnet.dk (H.E.); 5 Department of Cardiovascular Medicine, Lahey Hospital and Medical Center, Burlington, MA 01805, USA; Sachin.P.Shah@Lahey.org (S.P.S.); kamran.hayaan9189@gmail.com (H.K.); supria@gmail.com (S.B.); 6 Department of Cardiology, Henry Ford Hospital, Detroit, MI 48202, USA; MBASIR1@hfhs.org; 7 Department of Public Health (BIOSTIC), Aix-Marseille University, INSERM, IRD, APHM, UMR1252, SESSTIM, Hôpital de la Timone, 13005 Marseille, France; Julien.MANCINI@ap-hm.fr; 8 Department of Intensive Care Medicine, Aarhus University Hospital, 8200 Aarhus, Denmark; steffen.christensen@auh.rm.dk; 9 Department of Cardiology, Heart Center, Amsterdam Cardiovascular Sciences, Amsterdam UMC, University of Amsterdam, 1081 HV Amsterdam, The Netherlands; m.karami@amc.uva.nl (M.K.); j.p.henriques@amc.uva.nl (J.P.S.H.)

**Keywords:** Impella, cardiac arrest, refractory cardiac arrest, hemodynamic support device, cardiopulmonary resuscitation

## Abstract

Background: Impella CP is a left ventricular pump which may serve as a circulatory support during cardiopulmonary resuscitation (CPR) for cardiac arrest (CA). Nevertheless, the survival rate and factors associated with survival in patients undergoing Impella insertion during CPR for CA are unknown. Methods: We performed a retrospective multicenter international registry of patients undergoing Impella insertion during on-going CPR for in- or out-of-hospital CA. We recorded immediate and 30-day survival with and without neurologic impairment using the cerebral performance category score and evaluated the factors associated with survival. Results: Thirty-five patients had an Impella CP implanted during CPR for CA. Refractory ventricular arrhythmias were the most frequent initial rhythm (65.7%). In total, 65.7% of patients immediately survived. At 30 days, 45.7% of patients were still alive. The 30-day survival rate without neurological impairment was 37.1%. In univariate analysis, survival was associated with both an age < 75 years and a time from arrest to CPR ≤ 5 min (*p* = 0.035 and *p* = 0.008, respectively). Conclusions: In our multicenter registry, Impella CP insertion during ongoing CPR for CA was associated with a 37.1% rate of 30-day survival without neurological impairment. The factors associated with survival were a young age and a time from arrest to CPR ≤ 5 min.

## 1. Introduction

Sudden cardiac arrest (CA) is a major cause of death worldwide [1]. Survival following cardiopulmonary resuscitation (CPR) remains limited and the overall rate of survival to hospital discharge is about 10% for both in-hospital and out-of-hospital CA [2,3,4,5]. Despite advances in CPR, survival remains limited [6]. Following reports of a benefit of extracorporeal membrane oxygenation (ECMO) in patients with refractory CA, mechanical circulatory support (MCS) has emerged as a tool of potential interest [4,7]. Standardized protocols to better select patients who may benefit from ECMO and prevent futile use were proposed in the most recent guidelines [4,8]. Although ECMO support is associated with improved survival in selected patients, it is a resource-intensive therapy requiring a dedicated staff and therefore has a limited availability in centers without on-site ECMO [9,10]. The Impella CP device is a continuous flow pump inserted percutaneously into the left ventricle (LV), ensuring up to 3.5 L of blood flow per minute [11]. This device is available in cath-labs and could be quickly implemented during CPR. In recent years, case reports and monocenter registries have suggested a potential role for Impella CP in the setting of CA [12,13,14]. However, in these reports, the inclusion criteria and survival rates were variable, the sample size was limited, and predictors of success could not be determined. We therefore initiated an international multicenter registry of Impella implantation during on-going CPR for CA related to acute coronary syndrome (ACS), in order to investigate the outcome and the factors associated with survival.

## 2. Experimental Section

### 2.1. Participants and Informed Consent

The Impella CP registry is a retrospective multicenter registry from April 2014 to January 2020 of 35 patients who required Impella CP (Abiomed, Inc., Danvers, MA, USA) implantation during on-going CPR with manual or mechanical chest compression for CA presumably related to an ACS based on electrocardiography (ECG) and clinical features (ST segment elevation on ECG, shockable initial rhythm, or chest pain prior to the cardiac arrest). Only patients who had continuous on-going CPR when the Impella device was inserted were included. Patients with both in- or out-of-hospital witnessed CA were eligible. In out of hospital cardiac arrest (OHCA) situations, patients were transferred during ongoing CPR in hospital. If return of spontaneous circulation (ROSC) and hemodynamic stability were obtained before the Impella device was implanted and started pumping, cardiac arrest was not considered refractory and the patient was not included [4]. Patients without ongoing CA presenting with hemodynamic instability due to cardiogenic shock were not included in the analysis. The present retrospective registry conformed to the ethical guidelines of the 1975 Declaration of Helsinki and received institutional review board approval, and informed written consent was obtained from each patient before hospital discharge. When the patient was deceased, the consent was obtained from the family.

### 2.2. Study Procedure

The device was inserted during CPR in the catheterization laboratory via the femoral artery under vascular ultrasound guidance when possible and as specified by the manufacturer. Coronary angiography was conducted in all patients after Impella insertion and start. If required, revascularization was performed, depending on the clinical context. Chest compressions were continued until sufficient Impella flow could be achieved or until the patients was considered deceased. The neurological status was assessed using the cerebral performance category score (CPC score) collected from the medical health records. The weaning of the hemodynamic support was conducted according to each center’s protocols. Clinical data and survival at one month were collected retrospectively from electronic medical records in a dedicated database. Vascular complications were all complications requiring surgical care (including access site infection). Bleeding complications include all bleeding requiring transfusion. Time from arrest to CPR was defined as the time between collapse and the beginning of CPR (also called the “no flow time”). The CPR duration was the time of continuous manual or mechanical chest compressions (also called the “low flow time”). If a sustained and continuous ROSC (>30 min) was achieved, CA was not considered refractory and the patient was not eligible. When many short periods of unsustained ROSC were achieved, this time was deduced from the CPR duration.

### 2.3. Statistical Analysis

Statistical analyses were performed using IBM SPSS Statistics 20.0 (IBM Inc., New York, NY, USA). Continuous data are reported as the mean ± standard deviation. Categorical data are reported as absolute counts (percentages). Continuous data were compared using a Mann–Whitney test. Categorical data were compared using an χ^2^ test or a Fisher’s exact test. The Kaplan–Meier method was used to describe the probability of survival over time in the whole population. All tests were two-sided. Differences were considered to be statistically significant when the *p* value was less than 0.05. Figures were drawn using the GraphPad Prism 8 (GraphPad Software Inc., San Diego, CA, USA) software system.

## 3. Results

The registry included 35 patients from five different tertiary centers in four different countries, including the USA, Denmark, France, and The Netherlands, requiring ongoing CPR for CA.

### 3.1. Patients’ Characteristics

The main characteristics of the study population are described in Table 1. Patients had a mean age of 66 ± 9 years and the majority had suffered in-hospital CA (80.6%). The cardiac rhythm at the time of CA was a refractory ventricular arrhythmia in 65.7% of patients. The mean time from arrest to CPR was 3 ± 4 min. The mean time from CA to implantation of the Impella CP device and the delivery of blood flow was 46 ± 33 min. The duration of CPR for intra-hospital cardiac arrest (IHCA) patients was 39 ± 32 min, whereas the time of CPR for OHCA patients was 75 ± 40 min (*p* = 0.04). In all patients, an Impella CP device was used. The mean first in-hospital arterial pH and lactate levels were 7.0 ± 0.1 and 11.8 ± 3.8 mmol/L, respectively. Extended CPR was required following Impella start in 48.6% of patients. Coronary revascularization was performed in 85.7% of patients after device implantation. Four patients needed ECMO support after Impella insertion.

### 3.2. Outcome

**Figure 1:**
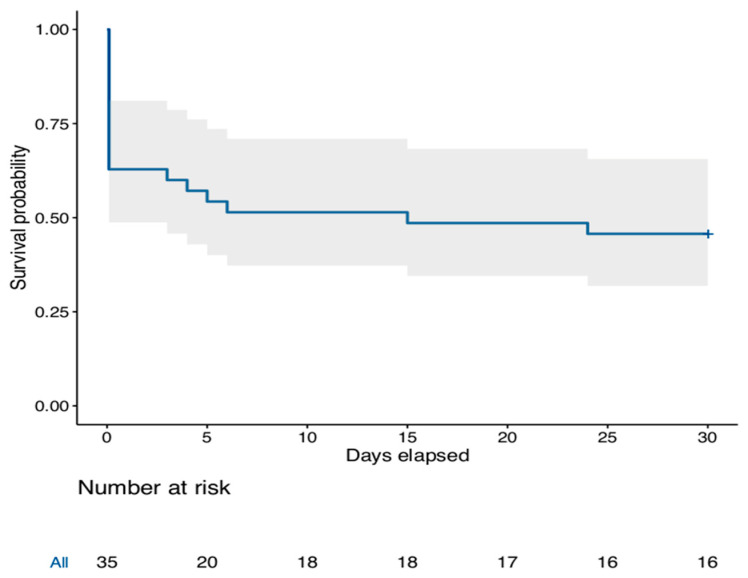
Kaplan–Meier curve showing the probability of survival over time.

Twelve patients (34.3%) died in the cath-lab and 23 (65.7%) survived the procedure and were transported to the intensive care unit. Following the procedure, all patients were under catecholamines (Table 1). The mean in-hospital length of stay was 17 ± 20 days (median 11 days IQR (1–26)). At 30-day follow-up, 16 patients (45.7%) were alive (Figure 1). Three patients out of the 16 survivors at 30-days had neurological sequelae (CPC score > 2). Overall, the 30-day survival rate without neurological sequelae (CPC score 1 or 2) was 37.1%. The cause of death was multi organ failure or refractory CA in all patients. Vascular and bleeding complications occurred in six patients (17.1%), of which two required vascular surgery, but none led to death. When considering survival at each center, we found a non-significant heterogeneity between participating hospitals (I^2^ = 43%, *p* = 0.149, Figure S1).

### 3.3. Factors Associated with Immediate and 30-Day Survival

**Figure 2:**
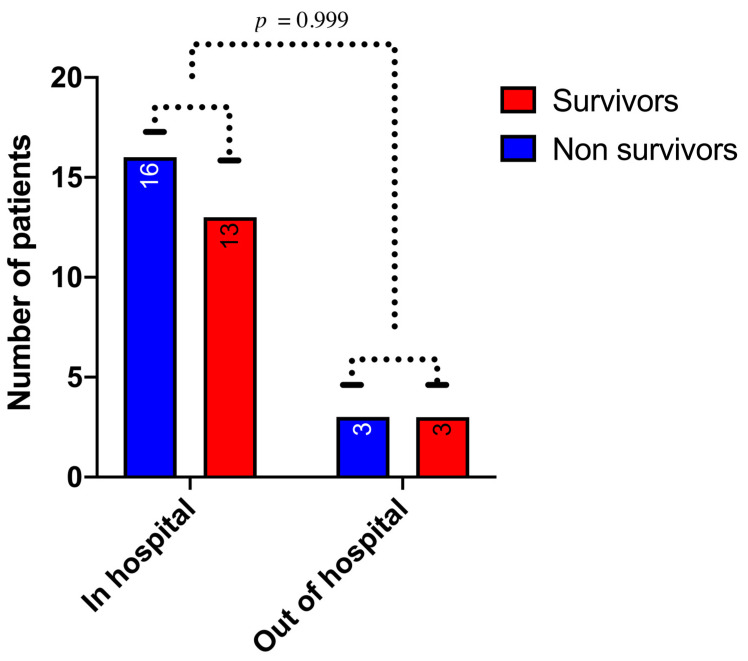
Relationship between the cardiac arrest location and 1-month survival (*p* value [Fisher’s exact test] = 0.999).

**Figure 3:**
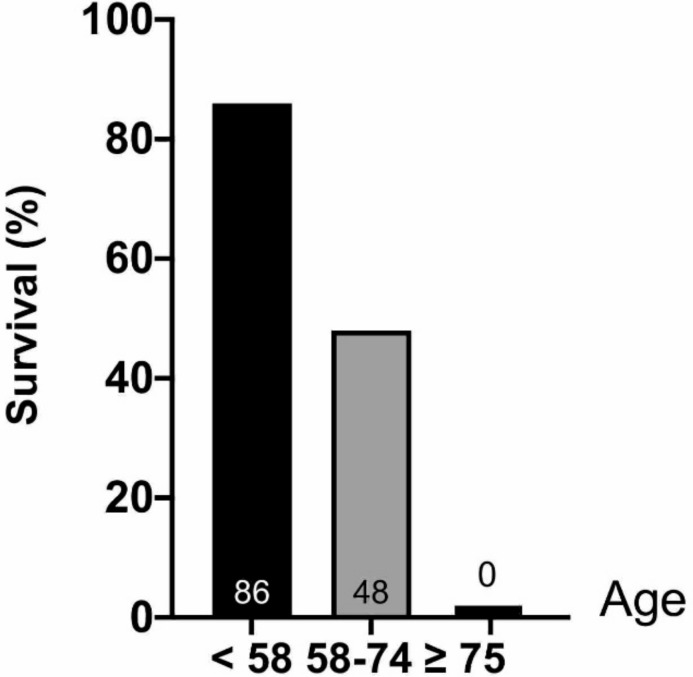
Relationship between age and 30-day survival.

**Figure 4:**
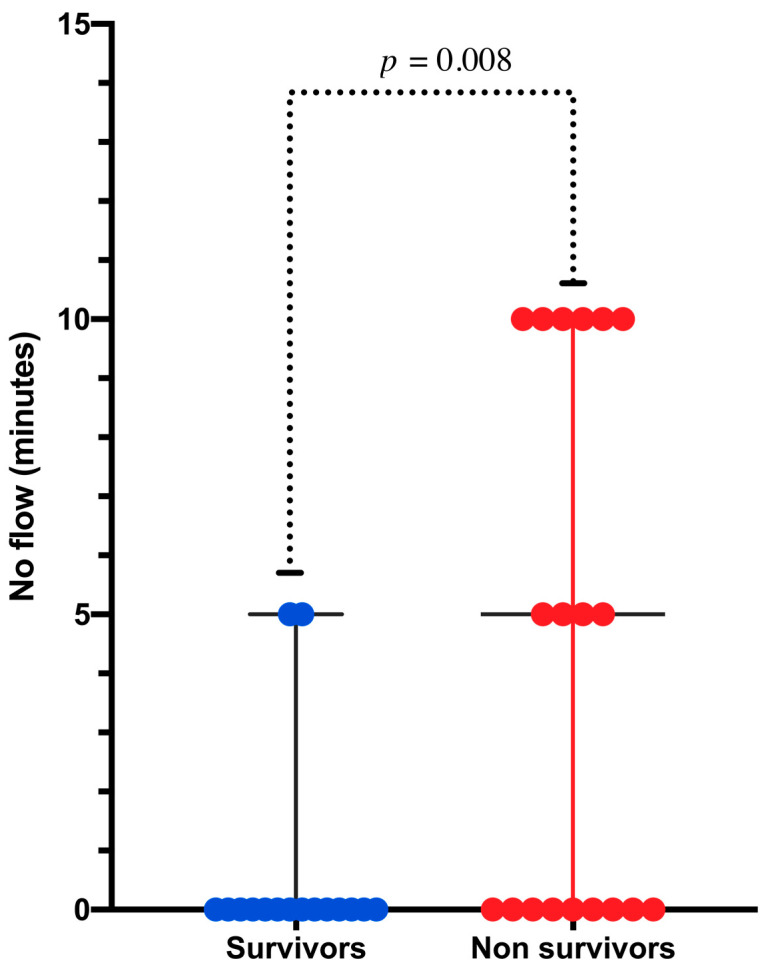
Scatter plot of time from arrest to cardiopulmonary resuscitation (CPR) (no flow time) in survivors and non survivors at 1 month (*p* value [Mann–Whitney] = 0.008).

As illustrated in Table 2, patients who survived the procedure were significantly younger (*p* = 0.02) and displayed a short time between CA and the initiation of CPR (*p* < 0.01). Although it did not reach statistical significance, there was a strong association between immediate survival and the presence of an initial shockable rhythm compared to pulseless electric activity or asystole (*p* = 0.059). Revascularization was not associated with immediate survival (*p* = 0.57). Regarding 30-day survival, we observed no difference in survival between in- and out-of-hospital CA (*p* = 0.99) (Figure 2). We observed a significant association between age and 30-day survival (*p* = 0.035). Among the seven patients over 75 years old, none survived to 30 days (Figure 3). The time between CA and CPR was also a factor associated with survival (*p* = 0.008) (Figure 4). Patients with a time from CA to CRP > 5 min did not survive. Again, although not significant, a trend toward a better 30-day survival was observed in patients with an initial shockable rhythm (*p* = 0.076).

## 4. Discussion

The Impella CP registry suggests that the early implantation of an Impella CP during ongoing-CPR for CA related to ACS is feasible and is associated with a 30-day survival rate of 45.7% and 37.1% without neurological sequelae. Previous monocenter studies have suggested that such an intervention could be successful. However, they had various inclusion criteria, involved a small sample size, and reported a large survival rate ranging between 5% and 50% [14,15]. The relatively high survival rate reported in our study was obtained despite the fact that the Impella was inserted as a salvage therapy during ongoing CPR without ROSC. However, because of the retrospective nature of the study and small sample size, these results should be considered preliminary and considered as a feasibility and safety analysis.

Although Impella is only an LV support device, it enabled successful resuscitation in a significant number of patients with ongoing CPR for CA. The feasibility and efficacy of only supporting the LV, with the Impella CP, during resuscitation in the present study are consistent with recent experimental and clinical evidence. Lotun et al. were able to resuscitate swine specimens in CA thanks to the combination of chest compressions and Impella, with favorable neurological recovery [16]. In their experimental model, the survival rate of Impella-facilitated resuscitation was superior to conventional CPR. Furthermore, in humans, consistent with our findings, previous reports have underlined the feasibility of LV support with only the Impella in CA [12,13,14,15].

In our study, the 1-month survival rate was 45.7% and 37.1% without neurological impairment. These results, although based on a limited number of patients, are promising and similar to those obtained with ECMO [17,18]. To date, few therapies have improved the outcome of CA [19]. ECMO is currently used in refractory CA based on promising registry data. Although there are no randomized studies, meta-analysis has suggested that ECMO utilization results in an overall survival rate of 22%, including 13% of patients with a good neurological recovery in refractory CA [7]. Furthermore, in another meta-analysis, ECMO was associated with a 13% absolute increase in 30-day survival compared to standard CPR [20]. However, the selection of patients is key, as demonstrated by the lack of overall benefit of ECMO compared to standard CPR in a propensity match analysis [21]. Lamhaut et al. reported that the appropriate selection of patients with CA for MCS is critical to preventing futile use [22]. Overall, these studies are in favor of a benefit of MCS in CA in selected patients. Accordingly, despite the relatively small sample size of our study, we were able to identify the duration of time from arrest to CPR and age as factors associated with survival. This result is original, since previous studies with the Impella could not assess factors associated with survival given their small sample size. Our findings are consistent with previous observations with ECMO [23] regarding these predictors, which are commonly used to select patients for MCS during CA [4]. Of note, our revascularization rate is close to those observed in the same setting in the literature [24]. Although the duration of CPR was not identified as a factor associated with survival in our study, it is well-recognized that a sustained time of CPR is associated with a poor outcome [25]. The lack of a significant impact of the duration of CPR in our study is probably related to the limited sample size. However, the factors associated with survival evidence in the present registry are useful for accurately selecting patients for Impella CP insertion during on-going CPR for CA and can help in the design of future trials in this field. Our results suggest that the implantation of Impella in patients over 75 years old or with a time from arrest to CPR > 5 min in the setting of ongoing CPR for CA may be futile.

In the setting of refractory CA, there are potential advantages of the Impella device over ECMO. First, Impella seems more available and quicker to insert by the interventional cardiologist than ECMO, which may ensure greater access to MCS for patients [26]. In addition, it reduces the time to full hemodynamic support and CPR duration, which are also critical for success in the setting of CA. Therefore, the Impella device may play a role in centers without ECMO or when the time to ECMO is expected to be long. Furthermore, the use of the Impella device may result in a lower rate of device-related vascular complications compared to ECMO [26,27]. Finally, ECMO-generated blood flow is continuous and retrograde, increasing ventricular stroke work and making this system efficient in terms of supporting peripheral organs, but responsible for LV overload [28,29]. On the other hand, Impella, despite a lower blood flow, is associated with several favorable properties of the heart by unloading the LV, limiting the infarct size, and promoting recovery [30]. However, there are no studies comparing ECMO and Impella in CA.

### Limitations

There are limitations to the present findings. First, the sample size is small, which may limit the generalizability of our results and prevented multivariate analysis for predictors of survival. Second, there is an obvious selective bias when using such an invasive and costly strategy for specific patients who may be considered as having a higher chance of survival and lower co-morbidities. Third, the majority of reported cases had good prognosis factors, such as in-hospital cardiac arrest and a limited period of time from arrest to CPR, and our result may not apply in a larger population. Additionally, the number of patients suffering OHCA is rather small and their time of CPR was longer than IHCA. Fourth, this is a retrospective observational study with inherent limitations and some clinical data were not available.

Nevertheless, the present study is the largest to date and suggests that in selected patients, Impella CP may be a lifesaving tool during CPR for CA related to an ACS. Prospective and larger studies are required to confirm these preliminary results, perform multivariate analysis for obtaining more reliable results, and help better define the patients likely to benefit from biventricular support rather than LV support alone during CPR for CA related to ACS.

## 5. Conclusions

In this international registry, in patients undergoing Impella CP insertion during on-going CPR for CA, the 30-day survival rate was 45.7% overall and 37.1% without neurological impairment. Impella CP insertion may be lifesaving in patients with CA younger than 75 years old with a time from arrest to CPR ≤ 5 min. Prospective studies are required to confirm these encouraging but preliminary data and should help to accurately define patient selection criteria.

## Figures and Tables

**Table 1:** Characteristics of the population.

**Variables**	**Entire Population (*n* = 35)**
Age (years)	66 ± 9/65 (59–72)
Male	28 (80.0)
**Location of cardiac arrest**	
IHCA	29 (80.6)
OHCA	6 (17.1)
**Initial rhythm**	
VF/VT	23 (65.7)
PEA/asystole	12 (34.3)
**CPR time courses**	
Time from arrest to CPR (min)	3 ± 4/0 (0–5)
CPR duration (min)	45 ± 36/30 (20–55)
Time between CA and Impella insertion (min)	46 ± 33/35 (26–60)
Extended CPR after Impella insertion	17 (48.6)
**Initial blood gas**	
Lactates	11 ± 3.8
Arterial pH	7.0 ± 0.1
**Use of catecholamines**	
Adrenaline	23 (65.7)
Dobutamine	12 (34.3)
Noradrenaline	23 (65.7)
Duration of Impella support (hours)	43.1 ± 48/24 (1–72)
Duration of inotropic support (hours)	151 ± 238.8/45 (1–200)
**Outcomes**	
Median hospital length of stay (days) mean ± SD	17 ± 20/11 (1–26)
Vascular complications	6 (17.1)
Immediate survival	23 (65.7)
Survival at 1-month	16 (45.7)

Values are the mean ± SD/median (IQR) for continuous data or n (%). Legend: CA: cardiac arrest; CPR: cardiopulmonary resuscitation; IHCA: intra-hospital cardiac arrest; OHCA: out-of-hospital cardiac arrest; PEA: pulseless electrical activity; VF: ventricular fibrillation; and VT: ventricular tachycardia.

**Table 2:** Univariate predictors of survival.

**Successful Resuscitation *n*, (%)**	**Deceased Patients (*n* = 12)**	**Survivors (*n* = 23)**	***p***
Age and mean ± SD	71 ± 7	63 ± 9	0.023
VF/VT	5 (41.7)	18 (78.3)	0.059
Time from arrest to CPR (min)	6 ± 5	1 ± 2	0.01
pH at initiation of CPR	7.0	7.02	0.487
Lactates level at initiation of CPR (mmol/L)	12.2	10.6	0.333
**1-month *n*, (%)**	**Deceased Patients (*n* = 19)**	**Survivors (*n* = 16)**	***p***
Age y mean ± SD	69 ± 8	62 ± 9	0.035
VF/VT	10 (52.6)	13 (81.2)	0.076
Time from arrest to CPR (min)	4 ± 4	1 ± 2	0.008
pH at initiation of CPR	7.04	6.98	0.298
Lactates level at initiation of CPR (mmol/L)	10.8	11.3	0.696

Values are the mean ± SD or *n* (%). Legend: VF: ventricular fibrillation, and VT: ventricular tachycardia.

## Supplementary Materials

The following are available online at https://www.mdpi.com/2077-0383/10/2/339/s1, Figure S1: Hetereogeneity between participating centers.
